# Tanning Misinformation Posted by Businesses on Social Media and Related Perceptions of Adolescent and Young Adult White Non-Hispanic Women: Mixed Methods Study

**DOI:** 10.2196/25661

**Published:** 2021-05-19

**Authors:** Megan Andreas Moreno, Marina C Jenkins, DeAnn Lazovich

**Affiliations:** 1 Department of Pediatrics University of Wisconsin-Madison Madison, WI United States; 2 Department of Epidemiology and Community Health University of Minnesota Minneapolis, MN United States

**Keywords:** adolescent, social media, tanning, technology, media

## Abstract

**Background:**

Indoor ultraviolet (UV) tanning is common and consequential, increasing the risk for cancers including melanoma and basal cell carcinoma. At-risk groups include adolescents and young adults, who often report beliefs about benefits of tanning. Adolescent and young adults are also among the most ubiquitous social media users. As previous studies support that content about tanning is common on social media, this may be a way that young women are exposed to influential content promoting tanning, including health misinformation.

**Objective:**

The purpose of this study was to evaluate health misinformation promoted by indoor tanning businesses via social media and to understand young women’s perceptions of this misinformation.

**Methods:**

This mixed methods study included (1) retrospective observational content analysis of indoor tanning salons’ content on Facebook over 1 year and (2) qualitative interviews with a purposeful national sample of 46 White non-Hispanic women, age 16 to 23 years, who had recently tanned indoors. We assessed experiences with tanning businesses’ posted content on social media through interviews. We used the constant comparative approach for qualitative analyses.

**Results:**

Content analysis findings included data from indoor tanning businesses (n=147) across 50 states, yielding 4956 total posts. Among 9 health misinformation topics identified, the most common was the promotion of UV tanning as a safe way to get Vitamin D (n=73, 1.5%). An example post was “Stop by Body and Sol to get your daily dose of Vitamin D.” Another misinformation topic was promoting tanning for health benefits (n=31, 0.62%), an example post was “the flu is not a season, it’s an inability to adapt due to decreased sun exposure…” A total of 46 participants completed interviews (age: mean 20 years, SD 2). Almost all participants (45/46, 98%) used Facebook, and 43.5% (20/46) followed an indoor tanning business on social media. Approximately half of participants reported seeing social media posts from tanning salons about Vitamin D, an example of a participant comment was “I have [seen that] a few times...” Among the participants, approximately half believed it was safe to get Vitamin D from indoor UV tanning; a participant stated: “I think it is a valid benefit to UV tanning.”

**Conclusions:**

Despite the low frequency (range 0.5%-1.5%) of social media posts promoting health misinformation, participants commonly reported viewing these posts, and their perceptions aligned with health misinformation. Health education campaigns, possibly using social media to target at-risk populations, may be an innovative approach for tanning prevention messages.

## Introduction

In 2009, the World Health Organization classified ultraviolet (UV) radiation-emitting tanning devices as Class I carcinogens [1]. Indoor UV tanning has been shown to increase the risk of developing squamous and basal cell carcinoma and melanoma, particularly among people who start at an early age or tan frequently [1-5]. Fortunately, indoor tanning has declined over the past decade among adolescents and young adults, who have traditionally represented a population with a high prevalence of this behavior [6,7]. However, among the highest adolescent risk group of White non-Hispanic females, indoor tanning rates remained above 10% in 2017, which is nearly twice the overall rate of tanning among adolescents [8,9].

Previous studies have identified several factors that influence an adolescent or young adult’s decision to pursue indoor tanning. These include beliefs about tanning contributing to beauty [10], social factors such as peer tanning behaviors [11], and positive attitudes and intentions toward tanning [12,13]. The role of media in promoting positive attitudes and intentions toward indoor tanning remains understudied. Previous studies have examined traditional media and found that watching reality television was associated with tanning [14] and that exposure to tan models in magazines promoted positive attitudes toward tanning [15]

In today’s digital world, messages promoting indoor tanning may be shared through social media [16], which has nearly ubiquitous use among adolescent and young adults [17]. Previous studies [18,19] support social media as a platform in which comparison to others’ appearances is positively associated with body dissatisfaction and even as a risk factor for eating disorders. Social media content related to tanning behavior is common, a previous study [20] examined Twitter and found 7.7 mentions of indoor tanning per minute, with only a small percentage mentioning health risks. While studies such as this have illustrated how users generate and share content about tanning on social media, less is known about the content shared by tanning businesses. Researchers in related fields, such as alcohol research, have hypothesized that the engagement and interaction that can occur with businesses via social media influence how marketing effects progress from awareness to encouraging behavior [21].

The spread of misinformation through social media can be particularly influential, as it is tied to emotions, identity, and one’s social network. For this study we define misinformation as inaccurate or false information. Indoor tanning salons’ business profiles on social media may increase exposure to misinformation among adolescent and young adults. The purpose of this mixed methods study was to evaluate health misinformation promoted by indoor UV tanning salons via social media and to understand adolescent and young adult’s perceptions of this misinformation.

## Methods

### Overview

This mixed methods study included social media content analysis and qualitative interviews. Content analysis was conducted on the social media platform Facebook between the dates May 1, 2015 and April 30, 2016. This data collection received an exemption for observation of public information from the Seattle Children’s institutional review board (15506 exempt).

Participant interviews were conducted after content analysis was complete in order to include observations from content analysis in the interview. These data were collected between May 8, 2017 and July 7, 2017. This data collection was approved by the Seattle Children’s institutional review board (15710).

### Social Media Content Analysis

#### Design

At the time of this study, Facebook was among the most popular sites used by the adolescent and young adult population [22]. Facebook also offered business profiles that allowed development of a free-standing multimedia profile with capacity to connect to and interact with users. Given its popularity among our target population and the robust content available for coding, this platform was selected for evaluation for this study.

#### Purposeful Sampling

The focus of this study was content posted on social media by businesses that provide indoor UV tanning. Our goal was to identify social media profiles created and maintained by indoor tanning salons. We focused on profiles that were popular among users and had both current and historical content to evaluate. Our goal was to evaluate 1 year of content retrospectively. For each of the 50 states, we evaluated 3 tanning business profiles to capture range and variation across businesses. We used purposeful sampling to identify profiles, a strategy used for description and investigation into social processes of particular groups.

#### Search Strategy

To identify potential profiles for evaluation, we conducted a search on Facebook for each of the 50 states using search terms in the form *“tanning salon” + [state]*, where *[state]* was replaced by each state name; we set the search criteria to deliver responses by relevance.

From the list of search results, we reviewed the first 10 business profiles listed, and from those profiles, we selected the 3 business profiles that had the largest number of followers.

We then evaluated each of the 3 profiles to ensure that they were publicly available profiles and that they prioritized UV tanning services. To confirm that the business prioritized UV tanning services, the business profile needed to meet a minimum of 2 of the 3 criteria: (1) include the word “tanning” in name of the business or business category selected on Facebook was “tanning salon,” (2) describe the provision of UV tanning services in the About Me section of the profile, and (3) have 25% or more of the posts by the profile owner in the past month refer to UV tanning. Additional inclusion criteria were that the business must have had the profile for at least 1 year (in order for us to be able to evaluate a full year of content) and that the business must have made at least 1 post during 2015 (to ensure that the profile was active during the coding period).

Facebook profiles that were labeled as unofficial pages were excluded: Facebook profiles have the option to indicate whether they are an official page sponsored by a busines or an unofficial page, which often represents an individual who is a fan or supporter of the business. Furthermore, because of our state-based approach to coding, tanning salon chains that extended to multiple states were excluded, though tanning chains located within single states were included.

If inclusion criteria were not met by a given profile, we selected the profile with the next highest number of followers. We developed a list of all salons that met inclusion criteria and the link to the Facebook page (so that the pages could be returned to later for coding).

#### Codebook Development and Variables

Through a previous study [23], we have created various codebooks containing keywords and image interpretation used in evaluating Facebook profiles for references to other health risk behaviors such as substance use and risky sexual behavior. Through a review of the literature [13,23,24] and pilot coding, we developed and tested a codebook focused on tanning-related health misinformation. We also recorded basic information about the tanning business, including the name, location, and number of followers.

#### Interrater Reliability

We conducted interrater agreement assessments at the beginning and end of the coding process. Interrater agreement ranged from 84% to 99% at the beginning to 91% to 100% at the end of coding across the health misinformation variables.

#### Procedure

Trained coders reviewed 1 year of content on each business social media profile. Assessing a full year of content allowed us to capture data to represent events (ie, spring break) and seasons. Pilot data collection illustrated that most tanning business profiles posted multiple times per day and frequently duplicated the same post during a given day or across a given month. Thus, our content analysis strategy was to evaluate 1 post per day, selected as the final post of that day, every third day of the month. This strategy allowed us to ensure that both weekdays and weekends were included in each month’s evaluations and to vary the day of the week evaluated over time. Furthermore, if that post was identical to the post evaluated from a previous day, that post was skipped and the prior post for that day was evaluated. This allowed us to diversify the content evaluated.

We recorded data for each selected post as follows: for text posts, we recorded verbatim text; for user-generated photos, we recorded a thorough description of the photo; and for popular public images, such as memes or other downloadable icons, we copied and pasted the image into the data set. We also recorded the date that the post was uploaded to the social media profile. Each social media post was considered the unit of analysis and was thus categorized and coded based on which constructs were represented in that post. Data were extracted to a customized database (FileMaker, Claris International) and saved into a secure data file that was password protected.

### Qualitative Interviews

#### Design

A qualitative approach was best suited to investigating young women’s experiences and perspectives [25]. The goal of this inquiry was to understand individual experiences and allow participants privacy in their responses to questions. Thus, individual interviewing was selected as the method.

#### Participant Sample and Recruitment

Given the qualitative approach, the goal sample size was 40 participants with experience with both indoor tanning and engagement with a tanning salon on social media. With purposeful sampling and this sample size, we estimated we could achieve theoretical saturation. We conducted purposeful recruitment to target participants who were among the demographic most likely to engage in tanning: White non-Hispanic females ages 16 through 23 years [26]. Our purposeful sampling also included focused recruitment efforts on participants who were likely to have engaged with indoor tanning salon content on social media. Thus, we recruited participants via Facebook advertisements. A Facebook advertisement was created and targeted to a national sample of women ages 16 through 23 years. The advertisement was posted for a total of 3 weeks. The advertisement described an interview study about indoor UV tanning experiences. Interested participants were directed to a website to complete eligibility screening to ensure they met inclusion criteria (experienced indoor tanning in the past year and were in our target demographic). These potentially eligible participants then underwent phone screening to confirm eligibility and complete informed consent. We obtained informed consent from participants over the age of 18 years and the parents of minors; We obtained informed assent from participants under the age of 18 years.

#### Interview Guide Development and Training

During interviews, we asked about perceptions and experiences viewing content from indoor tanning businesses on social media. Questions were designed to invite sharing of perspectives without judgment. Interview questions were designed to be semistructured and open-ended, allowing participants to expand their comments with follow-up prompts. Interview questions included asking participants about their indoor tanning experiences as well as their social media usage. Example questions included “In what ways do indoor tanning salons use social media?” Interviewers then provided a brief overview of the study findings regarding observations of health misinformation on social media. Participants were asked about thoughts, experiences, and reactions to this shared information.

Interviewer training involved reading training materials, observing standardized interviews, and conducting a minimum of 1 pilot interview prior to conducting interviews for this study. There were 2 interviewers for this study.

#### Data Collection

Interviews were conducted by phone. The interviewer confirmed that the participant was in a private and comfortable location prior to beginning the interview. Data were recorded in a customized online platform (FileMaker Pro) during the interviews. Interviews lasted between 20 and 40 minutes. Participants received a US $40 incentive upon completing the interview.

#### Analysis

Three investigators with experience in qualitative analysis were involved in the analysis process. The investigators utilized a constant comparative approach to categorize responses [25]. Two of the 3 investigators first individually reviewed all transcripts and then met to discuss data categorization. The goal of the first cycle was to collaboratively develop and apply a coding schema. The 2 investigators then independently coded 5 interviews (blinded to one another’s coding). After coding the transcripts, they reviewed the codes unblinded. All 3 investigators then met and discussed and reached consensus for any additions or revisions to coding categories. The third senior investigator served to resolve unclear areas or disagreements between investigators. The coding process was then applied to a second set of interviews with 2 investigators. The purpose of this second review was to evaluate reliability and validity of the initial classification criteria. After coding review, discussion and achieving consensus on the coding categories, the coding approach was applied to the remaining interviews using the same constant comparative approach.

## Results

### Social Media Content Analysis

Of a total of 147 indoor tanning business Facebook pages that were evaluated across 50 states, 3 businesses closed prior to coding initiation. This sample of indoor tanning business Facebook pages yielded 4956 posts. Among 8 health misinformation topics identified, the most common were posts promoting misinformation about Vitamin D (n=73, 1.3%). This misinformation typically focused on tanning as a safe and healthy way to get Vitamin D. The American Academy of Dermatology notes that tanning beds are not a safe way to get Vitamin D given the cancer risk [27]. Furthermore, since bulbs in tanning beds mostly emit UVA light, vitamin D can only be generated via UVB light. Thus, tanning beds do not provide sufficient exposure to create adequate doses of Vitamin D [27]. An example post was

Stop by Body and Sol to get your daily dose of Vitamin D.

Most indoor tanning businesses that displayed misinformation about Vitamin D did so infrequently. Many businesses had only 1 Vitamin D–related post within the 1-year sample, though 3 businesses had 5 or more of this category of posts.

A second category was misinformation about tanning as a medical treatment (n=31 posts, 0.6%), for example,

...the flu is not a season, it’s an inability to adapt due to decreased sun exposure…

Some of these posts were consistent with described inaccuracies in an investigative report provided to the US House of Representatives. These inaccuracies mainly center on promoting tanning as having health benefits or as a health treatment, there is no evidence to support tanning as a safe health treatment for illness including mental illness, inflammatory diseases, sleep problems or pain disorders [28]. Most businesses that displayed misinformation about tanning as a medical treatment had only 1 of these posts within the 1-year sample; there were no businesses with more than 3 posts of this type in the 1-year sample.

Another category of misinformation was promoting misinformation about the benefits of a “base tan” (n=29 posts, 0.5%) The posts often argued that a base tan would prevent sunburn or was a protective measure to take prior to a vacation. There is no evidence that a base tan is protective against sunburn or against cancer risks from sun exposure [27]. Most tanning businesses displayed only 1 of these posts in the 1-year sample, and many were posted in late winter, prior to spring break season. Table 1 displays the 8 categories of health misinformation, and Table 2 displays example social media posts.

**Table 1:** Health misinformation displayed on Facebook posts (N=4956) by businesses across 50 states over a 1-year period.

Misinformation category and number of posts		Businesses (N=147), n
**About Vitamin D (n=73)**		37
	1	26
	2	6
	3	2
	5	1
	9	1
	15	1
**Tanning as a medical treatment (n=31)**		26
	1	22
	2	3
	3	1
**Indoor tanning is equivalent to sunshine (n=31)**		18
	1	11
	2	4
	3	2
	9	1
**About base tan (n=29)**		14
	1	10
	2	3
	13	1
**Indoor tanning as safer than outdoor tanning (n=3)**		3
	1	3
**About health benefits of tanning (n=7)**		6
	1	5
	2	1
**About skin appearance (n=40)**		21
	1	15
	2	4
	3	1
	14	1
**About cancer (n=7)**		5
	1	4
	2	1

**Table 2:** Example posts.

Misinformation category	Posts
About Vitamin D	“Best supplementation of UV next to an IV” #VitaminD “D is for doping: Vitamin D”
Tanning as a medical treatment	“UVB from tanning is a safe treatment for acne, eczema and seasonal depression” “Link to article describing tanning as prevention for cancer, autism and depression”
Indoor tanning is equivalent to sunshine	“Tanning=sunshine” “Now serving sunshine”
About base tan	“A base tan will keep you safe from UV rays” “Start working on that base tan so you don’t burn at the beach”
Indoor tanning as safer than outdoor tanning	“Tanning indoor is taking responsibility for your tan. Prevent sunburn and using an indoor tanning lotion for best results!”
About health benefits of tanning	“Women who avoid sunbathing during the summer are twice as likely to die as those who sunbathe every day” “Getting enough sun optimizes 10% of our genes”
About skin appearance	“Tanning lotions will tighten your skin” “Tanning as ‘age rewinding’”
About cancer	“A great peer-reviewed journal article…but a little nerdy :) from the US National Library of Medicine and the National Institute of Health, showing that a continuous pattern of sun exposure appears NOT to increase risk of melanoma.” “According to a recent study the risk of skin cancer, particularly melanoma, decreases with proper tanning bed use”

### Qualitative Interviews

A total of 46 interviews were conducted (age: mean 20 years, SD 2) and 26 states were represented in our sample. Among our participants, 45 (97.8%) used Facebook, and 20 (43.5%) followed an indoor tanning salon on social media. Most participants (38/46, 82.6%) mentioned viewing indoor tanning salon posts on social media to find out about sales or special offers; fewer (3/46, 6.5%) noted that they learned tips related to tanning from these posts.

Approximately half (21/46, 45.5%) of participants acknowledged that indoor tanning businesses likely used social media to “draw people in” and influence tanning behavior. Some participants who followed tanning businesses on social media specifically noted that social media posts may normalize tanning behaviors or “promote tanning benefits.” Table 3 includes the most common topics that participants described viewing on indoor tanning salons’ social media pages.

Just over half (25/46, 54.3%) of participants expressed that social media influenced their friends, or people in general, to go tanning more often, and 52.2% (24/46) mentioned that social media influenced them personally to tan more. Some participants (4/46, 8.7%) stated that posts on social media by indoor tanning salons led them to see tanning as safer than the sun or the business as trustworthy.

As Vitamin D was the most common category of social media misinformation posts, we asked participants whether they had seen indoor tanning businesses post on social media about Vitamin D. Approximately half (20/46, 43%) of participants reported seeing social media posts from tanning salons about Vitamin D:

I have [seen that] a few times...

Participant

Among those participants, approximately half (25/46, 54%) believed it was safe to get Vitamin D from indoor tanning stating, for example,

I think it is a valid benefit to UV tanning.

Participant

**Table 3:** Qualitative interviews with White non-Hispanic adolescent and young adult women about indoor tanning and social media (n=46).

Topic			Participants (n=46), n (%)		Example quotations
**Type of content posted by indoor tanning salons on social media reported by participants**					
	Deals and sales	38 (82.6)		“They post $4 tan weeks, come tan for $5, promotions and deals mainly”	
	Lotions	12 (26.0)		“They post sales on lotion and promotions trying to get new people to come in, on Twitter for retweets they give away free lotion and tanning minutes”	
	Tanning tips	3 (6.5)		“…they also give tips for what is good for your skin and what isn't”	
**How tanning salons use social media**					
	Attract new customers	20 (45.5)		“They are trying to get customers or potential customers”	
	Showcase deals or promotions	18 (40.9)		“To promote brand recognition and specials they are running that month…They also promote employment for that salon. If you work at the salon, you get free tanning.”	
	Remind and/or motivate people to tan	7 (15.9)		“There is also the psychological aspect to it where they post motivations like, “everyone looks good tan” to try to get more people to start tan”	
	Sell lotions	6 (13.6)		“Advertising their prices and different lotions they have for their clients”	
	Promote tanning benefits	4 (9.1)		“They probably want to decrease the stigma in tanning to make it look more beneficial”	
	Target young people	3 (6.8)		“To reach their target market or to target those who are younger”	
**What role does social media play in you or your friends tanning**					
	Influences people to tan more	25 (54.3)		“I think it would make us tan more than we actually do want to or intend to”	
	Influence the participant to tan more	24 (52.2)		“It has made me want to tan. I used to be against, but then Facebook drew me into it and now I'm doing it”	
	Motivate to go tanning by deals	18 (38.1)		“If there's a deal more people will be inclined to do it, even people that don't regularly tan will go to take advantage of the deal”	
	Seeing friends post about tanning, not tanning salons, is influence to them	14 (30.4)		“When you see your friends on Facebook and they're tan it makes you want to be tan as well”	
	Motivate to go tanning by pictures of tan people	13 (28.3)		“I definitely see that it affects it. When you see people that are tan on social media you wish you were that tan”	
	Normalizes tanning	7 (15.2)		“I definitely thinking that seeing it a lot on social media makes it very normal. Since you see everyone else on social media, it makes it very acceptable”	
	Influences viewers to see tanning as safe	4 (8.7)		“Makes it seem like a safe thing to do. You don't think about the risks”	

## Discussion

### General

This mixed methods study included social media content analysis and qualitative participant interviews. We found an overall low frequency of social media posts promoting health misinformation (range 0.5%-1.5%); however, many (20/46,43.5%) of our participants actively followed tanning businesses on social media, and participants commonly reported remembering misinformation posts such as those promoting tanning as a safe way to get Vitamin D.

Our first finding was that social media posts related to health misinformation were uncommon. Most of the content posted by tanning businesses was related to other topics, such as deals or sales as described by our participants. These findings are consistent with a previous study examining hashtags related to tanning that found that most tanning salon posts were related to price reductions [29]. We found that the most common category of misinformation—indoor tanning as a safe way to get Vitamin D—comprised less than 2% of posts recorded across the year. It is important to clarify that our data did not represent that UV tanning could provide Vitamin D, but that posts described UV tanning as a safe way to achieve Vitamin D, which identifies these statements as clear misinformation as there is no evidence that the body can achieve adequate Vitamin D levels safely (given cancer risks) or adequately [27]. Furthermore, in many categories of health misinformation, most tanning businesses displayed only 1 post in that category across our 1-year evaluation period. Given these findings, it is even more striking that many participants reported recalling these posts. This finding suggests that these health misinformation posts, while uncommon, were memorable and may have been influential. Our findings from participant comments clearly support that the practice of following social media tanning businesses contributed to reminders to participants to go tanning, through deals and specials, and reminders about motivations to tan. It is also important to note that a social media platform is interactive, whereby users and businesses can interact bidirectionally. Thus, while the frequency of these health misinformation posts displayed by tanning businesses was uncommon, these posts can be shared or distributed across an individual’s own social media.

Our second finding was that, among our purposeful sample focused on those who engage in indoor tanning as well as social media, participants commonly engaged with social media related to tanning. As nearly half (20/46, 43.5%) participants followed a tanning business on social media, this supports the potential reach and influence that tanning salons have among young social media users. Participants described that indoor tanning salons’ social media pages provided them reminders, nudges, and information about special deals and sales. Participants described the influence of tanning-related social media on their own tanning attitudes, as well as on their behaviors. This finding suggests that, of the approximately 10% of young White females who continue to engage in indoor tanning, social media may be a viable platform to reach this at-risk population with education or intervention approaches.

### Limitations

Our study is not without limitations. This study focused on tanning businesses that were present on Facebook, and we did not examine other platforms such as Snapchat or Instagram. We were interested in how popular tanning businesses utilized social media to engage with customers. Thus, our purposeful sampling strategy prioritized selection of tanning businesses that were popular on social media (ie, by number of followers). We excluded tanning salon chains that extended across states, which allowed us to focus on individual businesses within states. However, tanning salons within chains may reach more viewers compared to individual businesses. Furthermore, our Facebook data were from 2015; however, the role of Facebook as a platform to connect to businesses remains relevant today, and no new laws or regulations governing tanning business content on social media have arisen since that time. Finally, our qualitative interviews were purposeful in order to target the at-risk population for indoor tanning, thus evaluating external validity of our sample was not appropriate for this study.

### Implications

Despite these limitations, our study has implications in the area of health misinformation. While the overall reduction in indoor tanning behaviors among women and adolescents [6,7] is a public health triumph, a significant at-risk group of White young women remain engaged in this health risk behavior [8,9]. Our study findings suggest that some of these women who tan are also connected to indoor tanning salons on social media. While misinformation may not be common or a significant motivator for adolescent and young adult tanning, social media connections between businesses and adolescents and young adults are very common and may provide ongoing engagement with and encouragement of tanning behavior, that is, this social media connection may foster ongoing relationships with tanning businesses and behavioral nudges to go tanning through deals and reminders about motivations to tan.

An initial strategy to prevent adolescents from following these businesses on social media may be to consider requiring *age-gating* for indoor tanning businesses. Age-gating would block access to indoor tanning social media pages for youth under age 18. The age-gating approach is currently used by alcohol companies on social media, and youth are supportive of applying this restriction to other businesses on social media [30]. Additional strategies may include creating regulations about health misinformation directed at indoor tanning businesses, similar to strategies used to limit health misinformation about tobacco and marijuana. Finally, strategies to reach at-risk women may include placing targeted educational campaigns on social media, similar to the Facebook advertisements that we used to recruit for this study, which were successful in identifying the population at risk for consequences of indoor tanning.

Our study focused on health misinformation messages shared on social media by tanning salons. However, the implications of our findings may contribute to a critical conversation about how health misinformation shared via social media may reach vulnerable populations and potential prevention strategies. First, age-gating may present a valid approach to limit youth access to content from businesses based on minimum age limits, such as alcohol, tobacco, e-cigarettes, marijuana, and tanning. Second, regulations that clearly define allowable messages on any medium (print, online, or social media) are critical to consider. Enhancing efforts around surveillance of social media would be an important part of such regulations. It is possible that funds from tax revenues related to these sale of these products could support regular and ongoing monitoring of industry compliance. Challenges to monitoring social media content include its potentially ephemeral nature, as well as the ability in social media to target content behaviorally, geographically, and temporally. While surveillance may not capture all businesses that post misinformation, for example, many of the businesses that posted Vitamin D misinformation did so only once during our data collection time period. However, ongoing surveillance may be likely to identify businesses that repeatedly post misinformation. For example, we found that 1 tanning business displayed 13 posts promoting misinformation about benefits of a base tan. Finally, our study illustrates that it is possible to create a sampling strategy to evaluate posts over time and to develop a codebook to identify content and achieve interrater reliability for such content. The content and keywords identified in this study may inform other social media surveillance methods using machine learning or automated text analysis for more widespread evaluation. It is also possible that partnering with adolescents and young adults who are often at the forefront of the learning curve for digital media may lead to creative prevention approaches.

## Abbreviations

UV: ultraviolet

## References

[1] El Ghissassi F, Baan R, Straif K, Grosse Y, Secretan B, Bouvard V, Benbrahim-Tallaa L, Guha N, Freeman C, Galichet L, Cogliano V, WHO International Agency for Research on Cancer Monograph Working Group (2009). A review of human carcinogens--part D: radiation. Lancet Oncol.

[2] International Agency for Research on Cancer Working Group on artificial ultraviolet (UV) lightskin cancer (2007). The association of use of sunbeds with cutaneous malignant melanoma and other skin cancers: a systematic review. Int J Cancer.

[3] Ferrucci L (2012). M., Indoor tanning and risk of early-onset basal cell carcinoma. J Am Acad Dermatol.

[4] Lazovich D, Vogel RI, Berwick M, Weinstock MA, Anderson KE, Warshaw EM (2010). Indoor tanning and risk of melanoma: a case-control study in a highly exposed population. Cancer Epidemiol Biomarkers Prev.

[5] Colantonio S, Bracken MB, Beecker J (2014). The association of indoor tanning and melanoma in adults: systematic review and meta-analysis. J Am Acad Dermatol.

[6] Guy GP, Watson M, Seidenberg AB, Hartman AM, Holman DM, Perna F (2017). Trends in indoor tanning and its association with sunburn among US adults. J Am Acad Dermatol.

[7] Holman DM, Jones SE, Qin J, Richardson LC (2019). Prevalence of indoor tanning among U.S. high school students from 2009 to 2017. J Community Health.

[8] Guy GP, Berkowitz Z, Tai E, Holman DM, Everett Jones S, Richardson LC (2014). Indoor tanning among high school students in the United States, 2009 and 2011. JAMA Dermatol.

[9] (2019). Skin cancer prevention progress report. Centers for Disease Control and Prevention.

[10] Mosher CE, Danoff-Burg S (2010). Addiction to indoor tanning: relation to anxiety, depression, and substance use. Arch Dermatol.

[11] Mosher CE, Danoff-Burg S (2005). Social predictors of sunscreen and self-tanning product use. J Am Coll Health.

[12] Mayer JA, Woodruff SI, Slymen DJ, Sallis JF, Forster JL, Clapp EJ, Hoerster KD, Pichon LC, Weeks JR, Belch GE, Weinstock MA, Gilmer T (2011). Adolescents' use of indoor tanning: a large-scale evaluation of psychosocial, environmental, and policy-level correlates. Am J Public Health.

[13] Lazovich D, Forster J, Sorensen G, Emmons K, Stryker J, Demierre M, Hickle A, Remba N (2004). Characteristics associated with use or intention to use indoor tanning among adolescents. Arch Pediatr Adolesc Med.

[14] Fogel J, Krausz F (2013). Watching reality television beauty shows is associated with tanning lamp use and outdoor tanning among college students. J Am Acad Dermatol.

[15] Dixon HG, Warne CD, Scully ML, Wakefield MA, Dobbinson SJ (2011). Does the portrayal of tanning in Australian women's magazines relate to real women's tanning beliefs and behavior?. Health Educ Behav.

[16] Hossler EW, Conroy MP (2008). YouTube as a source of information on tanning bed use. Arch Dermatol.

[17] Smith A, Anderson M (2018). Social media use in 2018. Pew Research Center.

[18] Cohen R, Blaszczynski A (2015). Comparative effects of Facebook and conventional media on body image dissatisfaction. J Eat Disord.

[19] Lee H, Lee HE, Choi J, Kim JH, Han HL (2014). Social media use, body image, and psychological well-being: a cross-cultural comparison of Korea and the United States. J Health Commun.

[20] Seidenberg AB, Pagoto SL, Vickey TA, Linos E, Wehner MR, Costa RD, Geller AC (2016). Tanning bed burns reported on Twitter: over 15,000 in 2013. Transl Behav Med.

[21] McClure Auden C, Tanski Susanne E, Li Zhigang, Jackson Kristina, Morgenstern Matthis, Li Zhongze, Sargent James D (2016). Internet alcohol marketing and underage alcohol use. Pediatrics.

[22] Lenhart A Pew Research Center.

[23] Moreno MA, Parks MR, Zimmerman FJ, Brito TE, Christakis DA (2009). Display of health risk behaviors on MySpace by adolescents: prevalence and associations. Arch Pediatr Adolesc Med.

[24] Bagdasarov Z, Banerjee S, Greene K, Campo S (2010). Indoor tanning and problem behavior. J Am Coll Health.

[25] Glesne C (1999). Becoming Qualitative Researchers. 2nd ed.

[26] Guy GP, Berkowitz Z, Everett Jones S, Holman DM, Garnett E, Watson M (2015). Trends in indoor tanning among US high school students, 2009-2013. JAMA Dermatol.

[27] 10 surprising facts about indoor tanning. American Academy of Dermatology Association.

[28] False and misleading health information provided to teens by the indoor tanning industry investigative report. Dermatology Nurses' Association.

[29] Ricklefs CA, Asdigian NL, Kalra HL, Mayer JA, Dellavalle RP, Holman DM, Crane LA (2016). Indoor tanning promotions on social media in six US cities #UVTanning #tanning. Transl Behav Med.

[30] Moreno MA, Gower AD, Jenkins MC, Kerr B, Gritton J (2018). Marijuana promotions on social media: adolescents' views on prevention strategies. Subst Abuse Treat Prev Policy.

